# CicArMiSatDB: the chickpea microsatellite database

**DOI:** 10.1186/1471-2105-15-212

**Published:** 2014-06-21

**Authors:** Dadakhalandar Doddamani, Mohan AVSK Katta, Aamir W Khan, Gaurav Agarwal, Trushar M Shah, Rajeev K Varshney

**Affiliations:** 1 International Crops Research Institute for the Semi-Arid Tropics (ICRISAT), Patancheru 502324, India

**Keywords:** Plant genomics, Database, Chickpea, *cicer*, Microsatellite, SSR

## Abstract

**Background:**

Chickpea (*Cicer arietinum*) is a widely grown legume crop in tropical, sub-tropical and temperate regions. Molecular breeding approaches seem to be essential for enhancing crop productivity in chickpea. Until recently, limited numbers of molecular markers were available in the case of chickpea for use in molecular breeding. However, the recent advances in genomics facilitated the development of large scale markers especially SSRs (simple sequence repeats), the markers of choice in any breeding program. Availability of genome sequence very recently opens new avenues for accelerating molecular breeding approaches for chickpea improvement.

**Description:**

In order to assist genetic studies and breeding applications, we have developed a user friendly relational database named the Chickpea Microsatellite Database (CicArMiSatDB http://cicarmisatdb.icrisat.org). This database provides detailed information on SSRs along with their features in the genome. SSRs have been classified and made accessible through an easy-to-use web interface.

**Conclusions:**

This database is expected to help chickpea community in particular and legume community in general, to select SSRs of particular type or from a specific region in the genome to advance both basic genomics research as well as applied aspects of crop improvement.

## Background

Chickpea belongs to the family Fabaceae of class dicots. Great importance has been attributed to chickpea in agriculture in view of its consumption as human food and livestock fodder. As per the FAO 2012 statistics [1], chickpea is grown in more than 50 countries and the production was approximately 11.3 million tons. India is the largest producer and it contributed to 67-70% in the world’s total production during 2009–2012. The two known types of chickpea, *kabuli* and *desi* are distinguished based on characteristics such as seed size, color and shape. *Desi* type is recognized by round dark seed coat, whereas, the *kabuli* type could be identified by bigger beige-colored round seed coat [2]. Chickpea is low in fat and provides dietary fibre, protein, dietary phosphorus and helps in the lowering of blood cholesterol [3]. As a member of family Fabaceae, it has the ability to increase the soil fertility by fixing the atmospheric nitrogen. In the context of crop improvement, the availability of the genomic sequence information opens the possibility of improving the crop production by developing the molecular markers for supporting breeding programs.

Molecular markers are specific sequence of DNA that identifies regions associated with trait of interest in the genome. A range of molecular markers namely restriction fragment length polymorphism (RFLP), random amplified polymorphism DNA (RAPD), amplified fragment length polymorphism (AFLP), simple sequence repeats (SSRs) also known as microsatellites and more recently, single nucleotide polymorphism (SNP) markers have become available in many crop species. SSRs, however, have been widely used in crop genetics and breeding applications [4]. For instance, SSRs have been used in determining hybrid purity, identifying genotypes, discovering genes linked to known markers and also enable an in-depth analysis of quantitative traits, allowing interesting alleles to be found in wild or cultivated germplasm [5].

SSRs are sequence blocks containing 1 to 6 nucleotide units repeated in tandem and tend to be highly polymorphic due to rapid mutation events. SSRs present advantages over other anonymous molecular markers like RAPD and AFLP as they occur randomly in a genome, allow identification of multiple alleles at single locus, and are co-dominant. These markers have been developed in number of crop species [6-8] for a broad range of applications such as genome mapping, genetic diversity studies and fingerprinting [4,9-11].

Recent advances in crop genomics enabled chickpea breeding community at a global scale to make significant improvements in the crop productivity by developing SSR markers from the various available resources like BAC-end sequences [12], transcriptome [13], SSR markers from SSR-enriched genomic library [10] and BAC libraries [14]. Recently, genome analysis of chickpea identified a total of 81,845 SSRs [15]. Primer pairs could be designed for 48,298 SSRs enabling them to be used as genetic markers. Given the huge number of SSRs, geneticists and breeders may be interested in selecting SSR markers from a specific genomic region. Therefore it is highly desirable to have SSR database for chickpea that enable chickpea community to select the SSR markers of choice. Such kind of SSR databases have been developed in some crops such as pigeonpea [16], sorghum, soybean, maize, rice [17] and cotton [18].

In view of above, this study reports a user friendly, comprehensive web based resource (CicArMiSatDB) detailing the information on SSRs present in the chickpea genome to facilitate use of SSRs as genetic markers in chickpea genetics and breeding applications. It is to be noted that the CicArMiSatDB not only contains the SSR markers for which primer pairs have already been reported but also highlight the ones (1,300 in total) which were validated in earlier studies.

## Construction and content

The list of chickpea SSRs [19] and genomic features [20] were collected and stored in relational database tables of PostgresSQL (v9.2.4). Importantly, genomic locations of validated SSRs, from earlier studies [2,10,12,13,21-27] were collected, and highlighted amongst the existing SSRs (Additional file 1: Table S1).

### Database architecture

**Figure 1:**
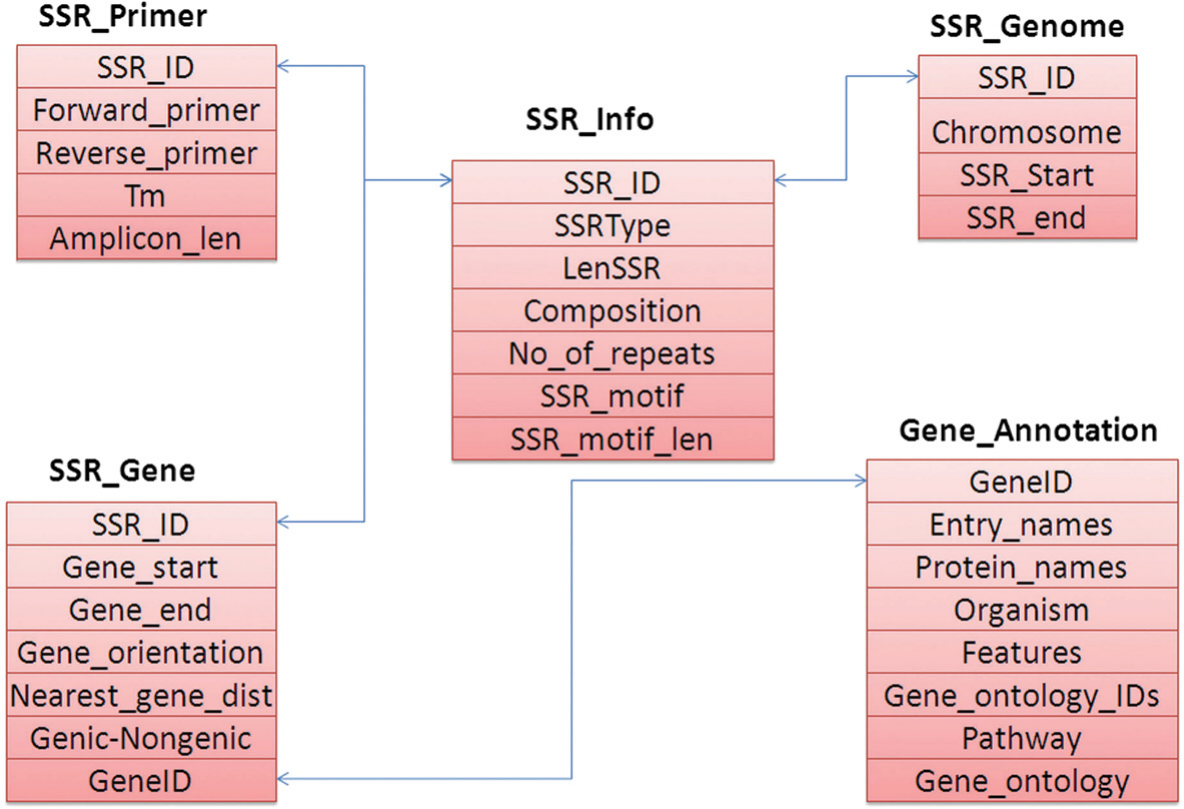
Database schema of CicArMiSatDB.

The information on SSRs was stored in five database tables (Figure 1). Each SSR was represented with a unique identifier called SSR_ID. The description of database tables is as follows.

1. **SSR_info table** contains SSRs that have been classified into simple and the compound SSRs based on the complexity of the motif. This table describes each SSR with the type of SSR, its length and the motif (2 to 6 nucleotides).

2. **SSR_primer table** provides the primer sequences which can be used for the amplification and information like amplicon size and melting temperature.

3. **SSR_genome table** provides information on the genomic coordinates of the SSR, and the classification (information on the location of SSR in the Pseudo molecules, contigs and scaffolds sequence).

4. The SSRs may be located either in coding or non-coding regions. The **SSR_gene table** contains classification of these SSRs into genic and non-genic categories based on their location inferred from the annotation file (gff). This table also includes the genomic coordinates, orientation of the genes and provides the nearest gene information along with the distance for the non-genic SSRs.

5. **Gene annotation table** contains the functional annotation of the genes such as gene name, symbol, protein function, organism, pathway information and Gene Ontology (GO) annotations.

### Implemented tools

1. **BLAST:**

To retrieve a marker and associated information, various search interfaces were included. Genome wide search for SSR markers was implemented by integrating BLAST [28,29] software into the database. The users may wish to search the database with a nucleotide sequence (e.g., gene of interest) to find the nearest genic and non-genic SSRs, both upstream and downstream to the sequence of interest, which could be used as candidate marker for further applications. To this end, BLAST search has been integrated into the database which enables the user to input multiple fasta format sequences to search for homologous sequences in chickpea genome. The genome coordinates of best hit from the search are resolved and screened within a window of 0.1 million bases (on both directions) to identify the nearest genic and non-genic SSRs in the chickpea genome.

2. **GBrowse:**

Generic genome browser (GBrowse) [30,31] was added to the database to visualize various genomic features like genes, CDS, SSRs etc. GBrowse enables visualization of the genomic features as well as comparison of SSRs in the database with the user provided SSRs in GFF [32] file format.

The database is designed by integrating software components such as PostgresSQL (v9.2.4): to store the data in tables; Apache web server (v2.22): to access the data using web interface with the help of PHP (v5.4) and jQuery (v2.0) library was used to ease the implementation of a user friendly interface to the database.

## Utility and discussion

Detailed analysis of chickpea genome through perl based MISA script [33] reported 48,298 SSRs [15]. The minimum numbers of repeat units observed in these SSRs were six for di-SSRs, five for tri-SSRs, four for tetra-SSRs, three for penta-SSRs and three for hexa-SSRs, with the longer loci generally having more alleles due to the greater potential for slippage [34].

**Figure 2:**
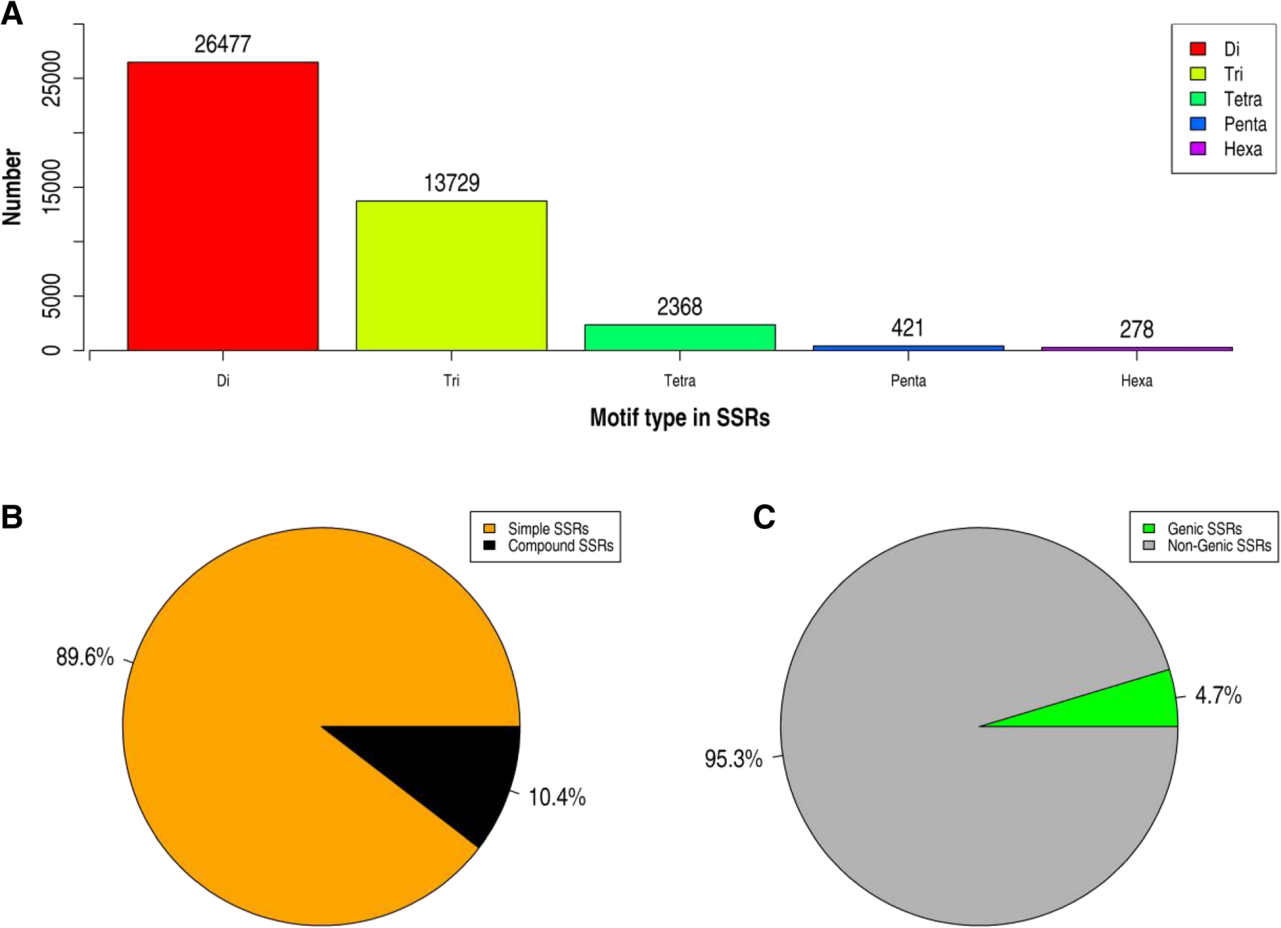
**Distribution and classification of SSRs in CicArMiSatDB. A)** Distribution of SSRs classified according to the motif repeats show the abundance of di and tri-nucleotide motifs. **B)** SSRs classified into simple and compound SSRs, simple SSRs constitute the majority of SSRs in chickpea genome. **C)** SSRs classified by their occurrence in genic and non-genic features show predominance of SSRs in non-genic regions.

Identified SSRs have been further classified in the database into simple and compound SSRs based on the complexity of the motif. Simple SSRs were found to be abundant in the genome constituting to 89.6% (43,273) of the total SSRs. In contrast, compound SSRs amount to only 10.4% (5,025) of the SSRs (Figure 2B). The most abundant simple SSR is di-SSRs (26,477) followed by the tri-SSRs (13,729), tetra-SSRs (2,368), penta-SSRs (421) and finally hexa-SSRs (278) (Figure 2A). The longest simple SSR was found to be hexa SSR with 49 repeating CAATTT motifs. The highest number of repeats was observed to be 132 in AT motif, (AT)_132_. Of the simple SSRs, the most frequently occurring motifs were AT (10,935, 41%) in di-SSRs, and AAT (1,820, 13.25%) in tri-SSRs.The SSRs classified based on genomic features (genic or non-genic) show that they occur predominantly in the non-genic regions (46,088, 95.42%) (Figure 2 C). On the other hand, the SSRs in genic regions were low (2,210, 4.57%) in number.

### Database as a tool to mine for known SSRs

The database search include simple and advance search with various options to explore the SSR information. Simple search will mine the database with any one of the listed options (see below) whereas advance search option could be used to mine SSRs by selecting two or more simple search criteria.

**Figure 3:**
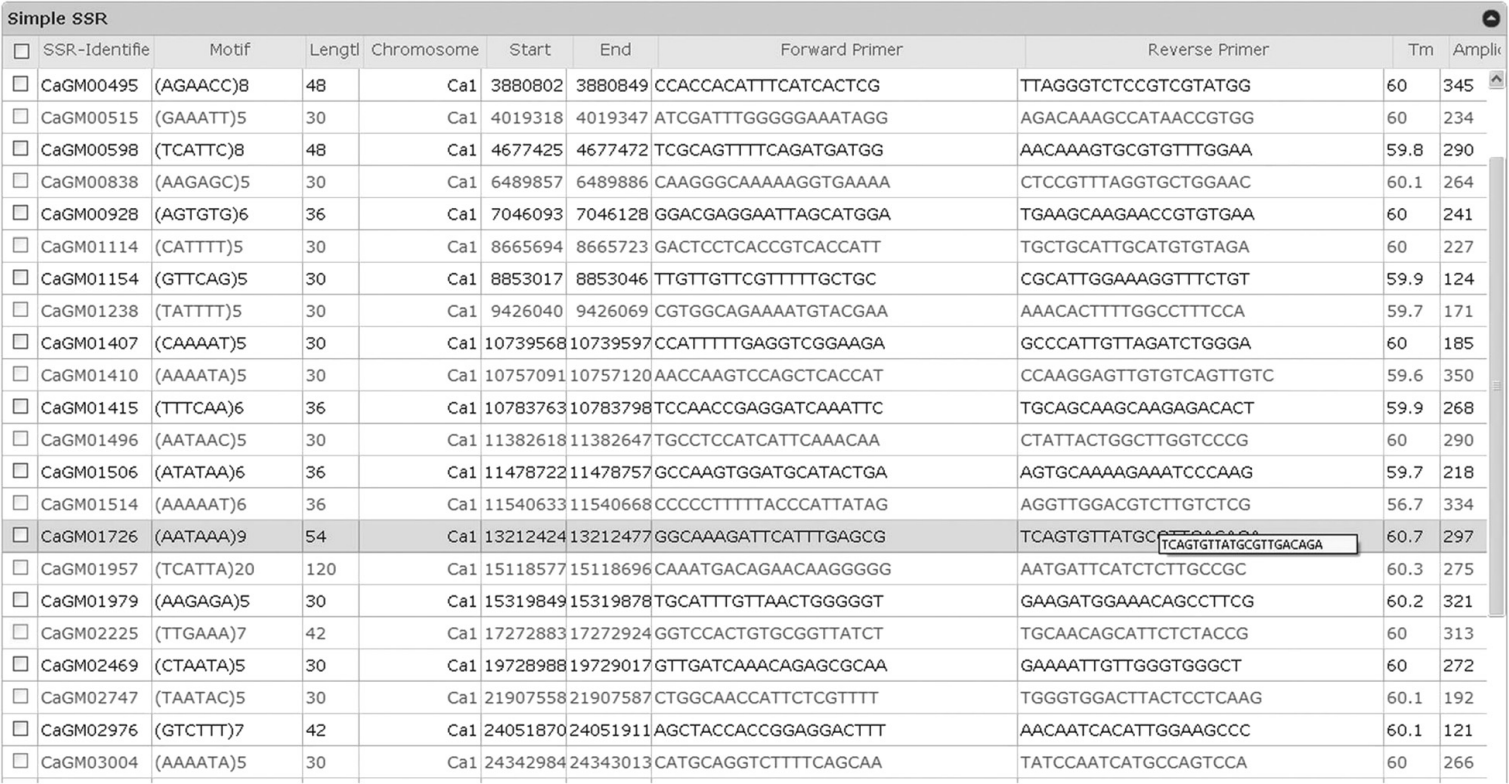
Simple SSR search result displaying chromosome co-ordinates, motif type, primers and other information.

The user can mine database using four options in the simple search as follows:

1. The type of the motif e.g. simple motif (classified into di, tri, tetra, penta and hexa repeats) and compound motif.

2. Based on the genomic locations of the SSRs, e.g. the ones found in regions like Contigs, Scaffolds and Pseudomolecules.

3. With a motif sequence of interest.

4. On the basis of genic and non-genic SSRs.Advanced search is implemented by combining 2 or more options of simple search. For example, one can search the simple SSR with the motif “TA” which is reported to be present in the pseudo-molecule number 5 (Ca5). The query result is tabulated with total number of SSRs found in the database along with genomic location as well as primers which could be used for amplification (Figure 3). Validated SSRs reported previously in the literature (1300 in number) have been highlighted with yellow color. Annotation information e.g. gene co-ordinates, orientation of the gene, gene symbols, function, UniProt ID, pathway information, gene ontology ID and gene ontology was also provided. However, in case of search for non-genic SSRs, similar information is displayed along with the details of nearest gene.

**Figure 4:**
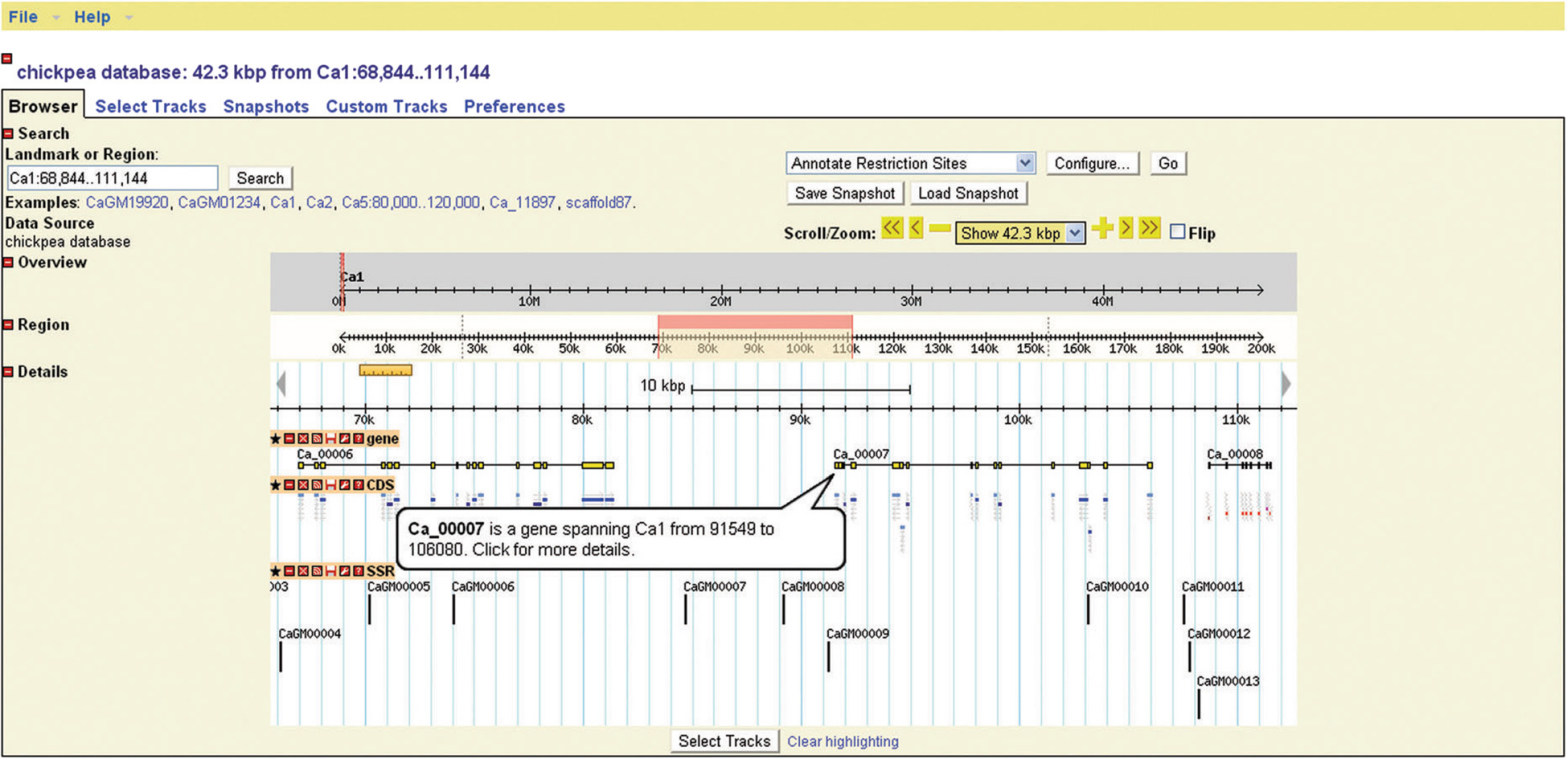
A GBrowse view displaying genomic features.

The search result could further be optionally customized. For example, one could restrict or filter the number of SSRs displayed within the range 25–100 results per page in a table. The table can be sorted depending on the unique SSR-ID and the chromosome in which SSR is present. BLAST search was integrated into the database to find the nearest genic and non-genic SSR available for the query sequence identified in the chickpea genome thereby enabling to discover linked SSRs. User can click on the marker information displayed on the BLAST result page to visualize the marker details in the configured genome browser (GBrowse). Additional details such as the sequence of the SSR could be obtained by clicking on the expanding icon (“+” symbol).GBrowse enables the user to graphically visualize different details (gene, CDS) present in the genome by extracting information from the GFF file. User can customize the tracks displayed by selecting the genomic features of their interest from the “select tracks” tab and type a search term or landmark into the text field at the top of the page. This fetches the region of the genome that spans the landmark, and displays it in an image panel called the “detailed view”. The detailed view consists of 3 horizontal tracks, each of which contains a particular type of sequence feature like gene, CDS and predicted SSR (Figure 4).

Further, one can upload set of custom markers in GFF format to GBrowse using “Add custom tracks” option of “custom tracks” tab. The users provided custom markers could be overlaid as track in GBrowse and visualize along with the database markers in order to confirm the novelty of SSRs.

We hope to include more features such as upstream/downstream elements, search for multiple SSRs based on BLAST search, and export of search results in excel sheet format as further updates to the database. We wish to add track containing information of the existing QTLs in the GBrowse also additional feature could be added to specify the physical location of the primer pairs on chickpea genome with the SSR repeat motif flanked by the primer pair.

## Conclusions

We have developed a comprehensive SSR database (CicArMiSatDB) for chickpea. The database includes powerful web-tools (BLAST and GBrowse) accessible with a user-friendly web interface to mine and filter the SSR markers. Advanced tools embedded in this database would help to query and visualize chickpea genome features. It classifies SSRs into genic and non-genic markers. Genic SSRs could be targeted for precise association with the trait of interest. The database is made openly accessible to the research community. It is developed to benefit the chickpea research in particular and legume research in general for both basic and applied studies.

## Availability and requirements

CicArMiSatDB has an open access and provides an integrated web interface to search and filter the simple sequence repeats in chickpea genome. This database is freely available online at http://cicarmisatdb.icrisat.org and works well with the CSS3 enabled browsers like Mozilla Firefox and the Google Chrome and Internet Explorer (9.0 or above).

## Abbreviations

BLAST: Basic local alignment search tool; QTL: Quantitative trait loci; CSS: Cascading style sheets; GFF: Generic feature format; PHP: Hypertext preprocessor.

## Competing interests

The authors declare that they have no competing interests.

## Authors’ contributions

Concept of the database was conceived by RKV. Design, development of database was done by DD, KM and RKV. Implementation and configuration of the GBrowse in the database was done by AWK and DD. GA and TS provided the validated SSR information from the literature. Suggestions and implementation guidance was provided by KM, TS, and RKV. DD together with RKV, AWK, KM, GA and TS prepared the MS and RKV finalized the manuscript. All authors read and approved the final manuscript.

## Supplementary Material

Additional file 1: Table S1 List of published SSR markers included in the CicArMiSatDB database. Click here for file
